# Knowledge and Awareness of Myocardial Infarction Symptoms and Appropriate Emergency Response Among the General Adult Population in Jeddah, Saudi Arabia: A Cross-Sectional Study

**DOI:** 10.7759/cureus.115397

**Published:** 2026-08-29

**Authors:** Yasser B Shahid, Abdullah M Al Garni, Majed A Jawa, Ahmad M Al Jifri, Abdulaziz M Sultan, Anas K Halawani

**Affiliations:** 1 Internal Medicine Department, Ibn Sina National College for Medical Studies, Jeddah, SAU

**Keywords:** cross-sectional study, emergency response, myocardial infarction, public awareness, saudi arabia, symptom recognition

## Abstract

Background and objective

Delayed recognition of myocardial infarction (MI) symptoms is a preventable driver of cardiovascular mortality, yet the extent to which the public in Jeddah, Saudi Arabia, can identify these symptoms and respond appropriately in an emergency has not been well studied. This study aimed to assess awareness of MI symptoms and the appropriate emergency response among adults in Jeddah, and to determine which sociodemographic and health-related factors are associated with this awareness.

Methods

In this descriptive cross-sectional study, 281 adults aged 18 years and older in Jeddah completed a self-administered online questionnaire covering sociodemographic and health-related characteristics, recognition of six typical and atypical MI symptoms (yielding an awareness score ranging from 0 to 6), and knowledge of the correct emergency response. Correct recognition of a symptom was scored as an affirmative “Yes” response, with “No” and “Don’t know” both scored as non-recognition. Associations between awareness and sociodemographic variables were tested using chi-square tests, independent-samples t-tests, and one-way analysis of variance (ANOVA), with statistical significance set at p < 0.05.

Results

The sample was 60.1% female, and the mean awareness score was 4.02 ± 2.02 out of 6. Shortness of breath (76.9%) and chest pain (75.8%) were recognized far more often than nausea or vomiting (50.9%) and jaw or neck pain (54.8%). At the participant level, 176/281 (62.6%) correctly recognized all three typical symptoms, 43/281 (15.3%) recognized two, 21/281 (7.5%) recognized one, and 41/281 (14.6%) recognized none. Nearly all participants (94.0%) knew that calling an ambulance was the appropriate first step, but only 26.7% incorrectly identified 997 as the national emergency number, while the majority (66.5%) correctly identified 911. Two-thirds of participants (66.9%) fell into the high-awareness group (≥ 4/6 symptoms recognized), but this did not differ significantly by gender (p = 0.506), education (p = 0.318), or age group (p = 0.914); mean awareness scores likewise showed no significant differences across these same groups (p = 0.379, 0.633, and 0.799, respectively).

Conclusions

Adults in Jeddah generally recognized classic MI symptoms and knew the correct emergency response, but recognition of atypical symptoms was lower and was not associated with the sociodemographic factors examined. Public health campaigns that specifically address atypical presentations, particularly nausea and jaw or neck pain, may be warranted for the population as a whole rather than targeting any single subgroup.

## Introduction

Cardiovascular disease, including myocardial infarction (MI), remains the leading cause of mortality worldwide, accounting for an estimated 18 million deaths each year [1,2]. Clinical outcomes following MI are strongly time-dependent [3]. The interval between symptom onset and the initiation of appropriate medical care, commonly termed patient delay, is one of the most important modifiable determinants of survival and long-term prognosis. Prompt recognition of MI symptoms and immediate activation of emergency medical services can substantially reduce myocardial damage and improve outcomes, whereas delayed recognition contributes directly to preventable morbidity and mortality.

Classic MI symptoms, such as chest pain and shortness of breath, are generally well recognized by the public. In contrast, awareness of atypical symptoms, including nausea or vomiting, dizziness, and jaw, neck, or back pain, tends to be considerably lower, even though these presentations are common, particularly among women, older adults, and patients with diabetes [4-6]. Meta-analytic evidence indicates that women are less likely than men to experience and recognize classic chest pain during an MI, which may partly explain sex-related differences in diagnostic delay and in-hospital outcomes [4,7].

Evidence on the sociodemographic determinants of MI awareness has been inconsistent. Some studies report that higher educational attainment and income are associated with greater awareness of MI symptoms and risk factors, while others report no such association [8,9]. Similarly, findings on gender differences in awareness have varied across populations, with some studies reporting lower awareness among males and others finding no significant difference [8-11]. Knowledge of the correct immediate response to suspected MI, such as calling emergency medical services rather than self-managing symptoms or seeking non-urgent care, has also been shown to vary considerably between populations and settings [10-14].

Within Saudi Arabia, several regional studies have assessed public knowledge of MI symptoms and risk factors, generally reporting moderate to good awareness of classic symptoms, alongside persistent gaps in the recognition of atypical presentations and knowledge of appropriate emergency responses [8,10-22]. Nationally, the Saudi Acute Myocardial Infarction Registry (STARS-1) has similarly highlighted delays and gaps in guideline-adherent emergency care among patients ultimately diagnosed with MI [23,24], reinforcing the importance of public-facing symptom education alongside health-system-level interventions [23].

Modifiable behavioral and metabolic risk factors are estimated to account for the majority of MI risk globally [25], underscoring the value of public awareness of both risk factors and warning symptoms. However, evidence specific to the general adult population of Jeddah, one of the Kingdom’s largest urban centers, remains limited, and the sociodemographic and health-related determinants of awareness in this setting have not been well characterized [26-30]. Given ongoing public health efforts in Saudi Arabia to reduce cardiovascular mortality, including initiatives aligned with Saudi Vision 2030 [23], up-to-date local evidence is needed to inform effective, targeted awareness campaigns. This study therefore aimed to evaluate the level of awareness of MI symptoms and the appropriate emergency response among the general adult population in Jeddah, Saudi Arabia, and to identify the sociodemographic and health-related factors associated with this awareness, in order to support the design of targeted public health interventions.

The study's broader objectives were as follows: (1) The study seeks to determine the proportion of participants who correctly recognize all three predefined major typical MI symptoms: chest pain, shortness of breath, and pain or heaviness in the left arm. Recognition of each symptom will be defined as an affirmative “Yes” response to the corresponding questionnaire item, while “No” and “Don’t know” responses will be considered non-recognition. The study will also describe the distribution of participants who recognize 0, 1, 2, or all 3 typical symptoms. (2) In addition, the recognition rates of the three assessed atypical MI symptoms, jaw or neck pain, nausea or vomiting, and sudden excessive sweating, will be determined based on the proportion of participants (N = 281) who report “Yes” to each corresponding questionnaire item. (3) The study will further identify the most commonly reported appropriate first action following the onset of MI symptoms as a behavioral indicator of awareness of the appropriate emergency response. (4) Finally, the study will examine the association between awareness scores and selected sociodemographic characteristics, including age, gender, and education, as well as health-related factors such as chronic disease status and family history of MI. This analysis will help determine whether gaps in MI awareness are concentrated among specific population subgroups, which may support targeted educational campaigns, or are broadly distributed across the population, thereby supporting population-wide awareness initiatives.

## Materials and methods

Study design and setting

A descriptive, cross-sectional study design was employed. The study was conducted among the general adult population of Jeddah, Saudi Arabia, between February and May 2026.

Study population

Adults aged 18 years or older, who were residing in Jeddah and able to understand and complete the study questionnaire, were eligible for inclusion. Healthcare professionals and healthcare students were excluded because of their specialized medical knowledge, as were individuals who did not provide informed consent.

Sample size and sampling technique

The target sample size was calculated using the Cochran formula for a single population proportion:

$\displaystyle{\displaylines{n = Z&sup2;P(1&minus;P) / d&sup2;}}$

Using Z = 1.96 (95% confidence level), P = 0.5 (the conservative, maximum-variance estimate adopted in the absence of prior local awareness data), and d = 0.05 (5% margin of error), the calculated sample size was n = 384.16, rounded up to a target of 385 individuals to achieve a 95% confidence level and a 5% margin of error. Participants were recruited online through social media platforms using a snowball sampling technique [26], in which initial respondents were asked to share the questionnaire with other eligible adults in their network. A total of 281 valid responses were obtained and analyzed, corresponding to 73.0% of the calculated target sample. This shortfall is addressed explicitly in the Limitations section, including its quantitative effect on the achieved margin of error and the statistical power of subgroup comparisons.

Data collection tool

Data were collected using a self-administered, structured, pre-tested questionnaire developed in both Arabic and English. The questionnaire comprised three parts. Part 1 captured sociodemographic and health-related data, including age, gender, educational level, marital status, chronic disease status, and family history of MI. Part 2 assessed awareness of MI symptoms using a checklist covering three typical symptoms (chest pain, shortness of breath, and pain or heaviness in the left arm) and three atypical symptoms (jaw or neck pain, nausea or vomiting, and sudden excessive sweating), with response options of “Yes,” “No,” or “Don’t know.” A response of “Yes” was scored as correct recognition of that symptom; “No” and “Don’t know” were both scored as non-recognition. Part 3 assessed awareness of the appropriate emergency response, including the correct first action upon witnessing MI symptoms, perceived danger of delaying hospital presentation, perceived safe waiting time, preference for ambulance transport over private transport, and knowledge of the national emergency number in Saudi Arabia. All Part 2 and Part 3 items were self-reported by the participants and were not assessed by investigators or clinicians. Data were collected between February and May 2026 from adults residing in Jeddah, Saudi Arabia, who were recruited online, with the aim of characterizing baseline public awareness to inform future public health messaging.

Study variables

The primary outcome variable was an overall awareness score, computed for each participant as the number of the six listed MI symptoms (chest pain, shortness of breath, left-arm pain, jaw/neck pain, nausea or vomiting, and sudden excessive sweating) correctly recognized, yielding a possible range of 0 to 6. A secondary variable, the typical-symptom sub-score (range 0-3), was computed as the number of the three typical symptoms (chest pain, shortness of breath, and left-arm pain) correctly recognized, to directly address Objective 1. For bivariate analysis, participants were dichotomized into high awareness (recognizing ≥ 4/6 symptoms) and low awareness (recognizing ≤ 3/6 symptoms). Independent variables included age group, gender, educational level, marital status, chronic disease status, and family history of MI.

Statistical analysis

Data were analyzed using IBM SPSS Statistics (IBM Corp., Armonk, NY). Descriptive statistics (frequencies and percentages) were used to summarize sociodemographic characteristics and the prevalence of awareness for each symptom and appropriate action; the mean and standard deviation (SD) were used to summarize the overall awareness score. The chi-square test was used to examine associations between categorical sociodemographic variables and dichotomized awareness level (high/low). Independent-samples t-tests and one-way analysis of variance (ANOVA) were used to compare mean awareness scores between and across groups. A p-value < 0.05 was considered statistically significant.

Ethical considerations

The study protocol (“Knowledge and Awareness of Myocardial Infarction Symptoms Among the General Adult Population in Jeddah, Saudi Arabia,” Protocol ID: 045MP/CR19022026) received expedited approval from the Institutional Research Review Board (IRRB) of Ibn Sina National College for Medical Studies (Reference No. IRRB-15-02032026, approved February 19, 2026), in accordance with the Implementing Regulations of the Law of Ethics of Research on Living Creatures in the Kingdom of Saudi Arabia. Participation was voluntary, and informed consent was obtained electronically from all participants prior to questionnaire completion. Participants were informed of the study purpose and their right to withdraw at any time, and no personally identifying information was collected.

## Results

Of the 281 participants, 169 (60.1%) were female, and 112 (39.9%) were male (Table 1, Figure 1). Just over half of the participants were married (52.3%), and nearly half held a bachelor’s degree (49.5%), followed by high school education (26.7%), a diploma (14.2%), and postgraduate education (9.6%) (Table 1, Figures 1-3). Regarding age, 35.6% of participants were aged 18-29 years, 35.6% were aged 30-39 years, 18.1% were aged 40-49 years, and 10.7% were aged 50-60 years (Table 2, Figure 4).

**Table 1:** Sociodemographic characteristics of the participants (N = 281)

Variable	Category	N	%
Gender	Male	112	39.9
	Female	169	60.1
Marital status	Married	147	52.3
	Single	134	47.7
Educational level	High school	75	26.7
	Diploma	40	14.2
	Bachelor's	139	49.5
	Postgraduate studies	27	9.6
Total		281	100

**Figure 1:**
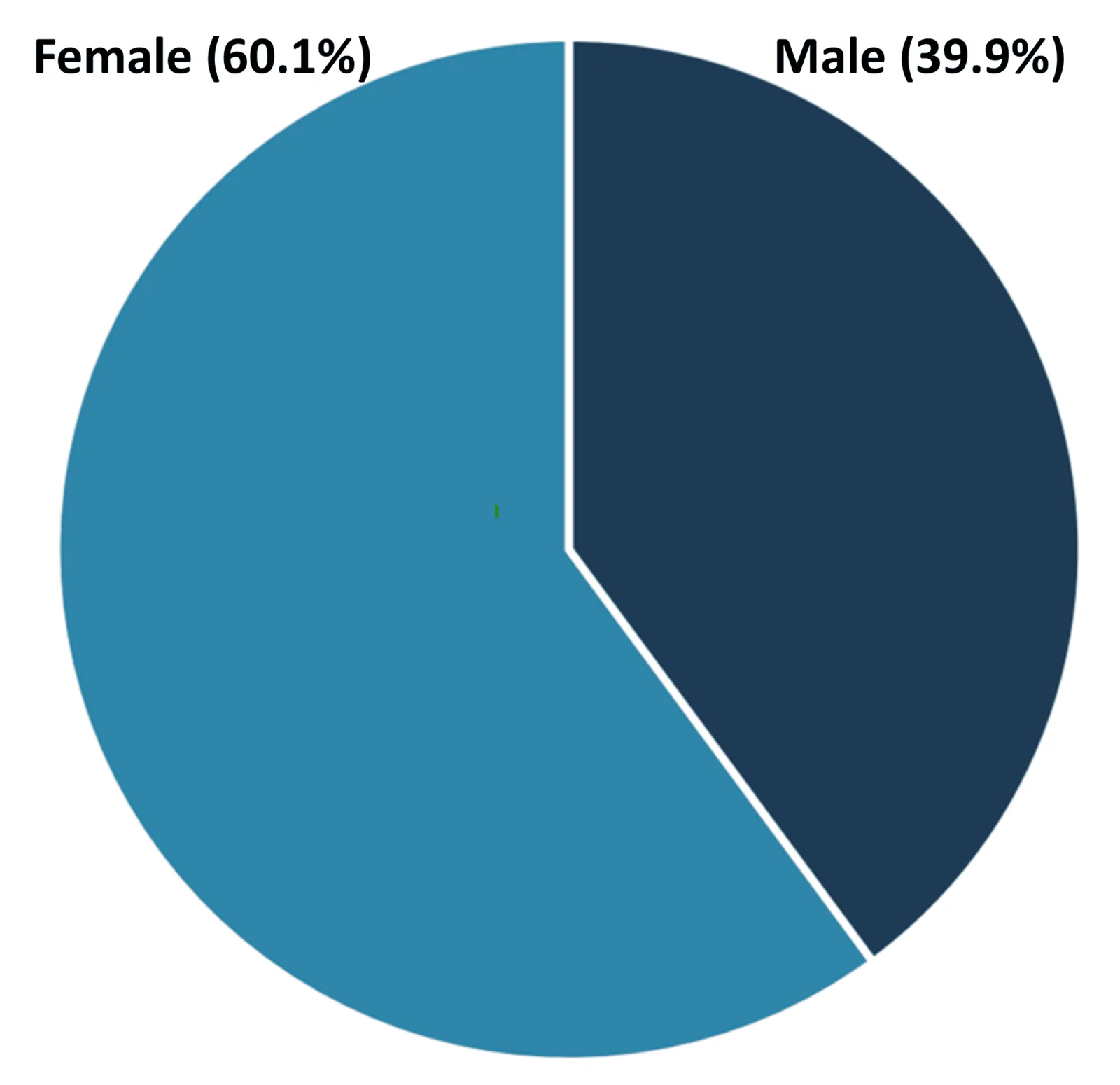
Gender distribution of the participants (N = 281)

**Figure 2:**
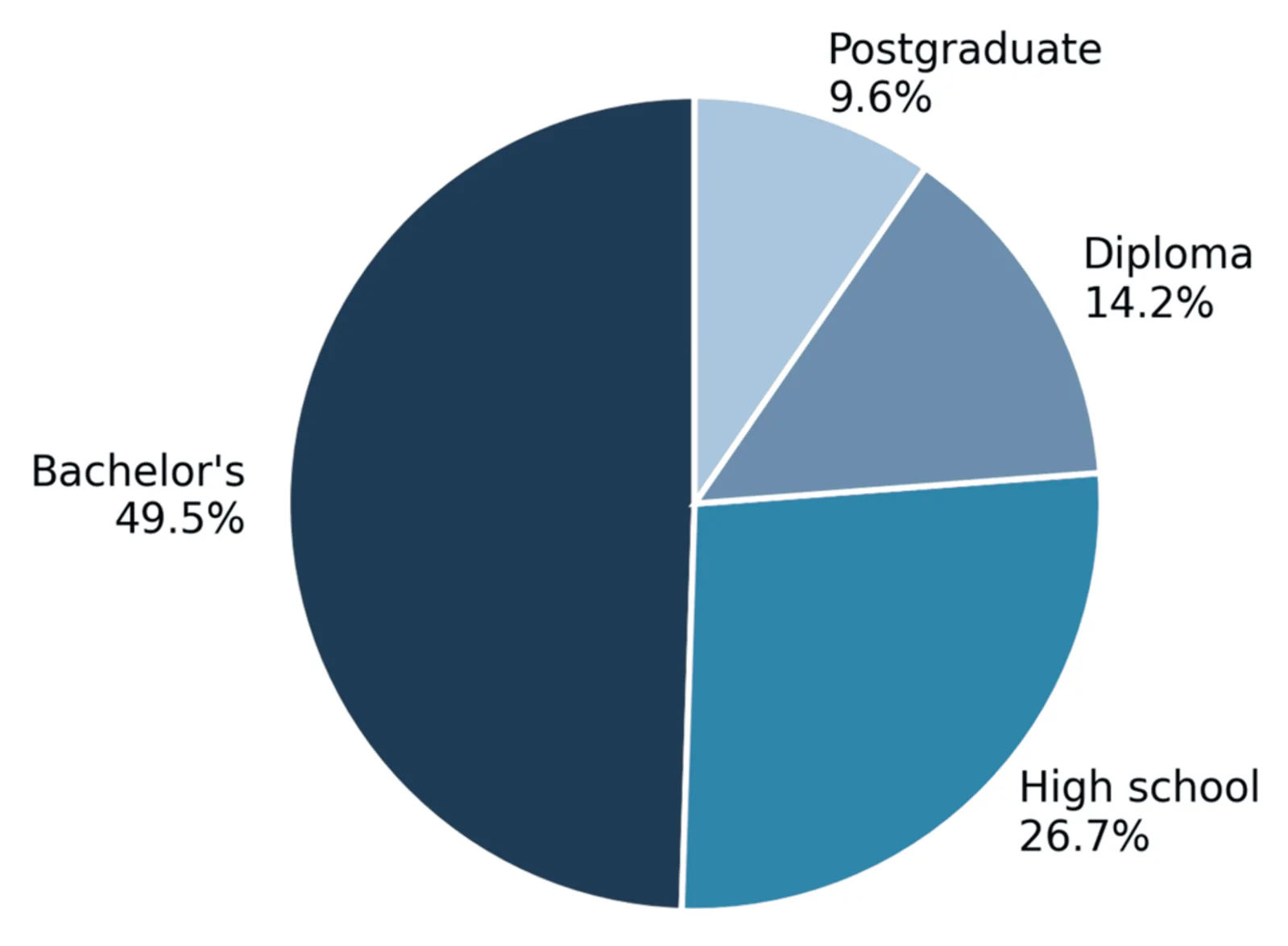
Educational level of the participants (N = 281)

**Figure 3:**
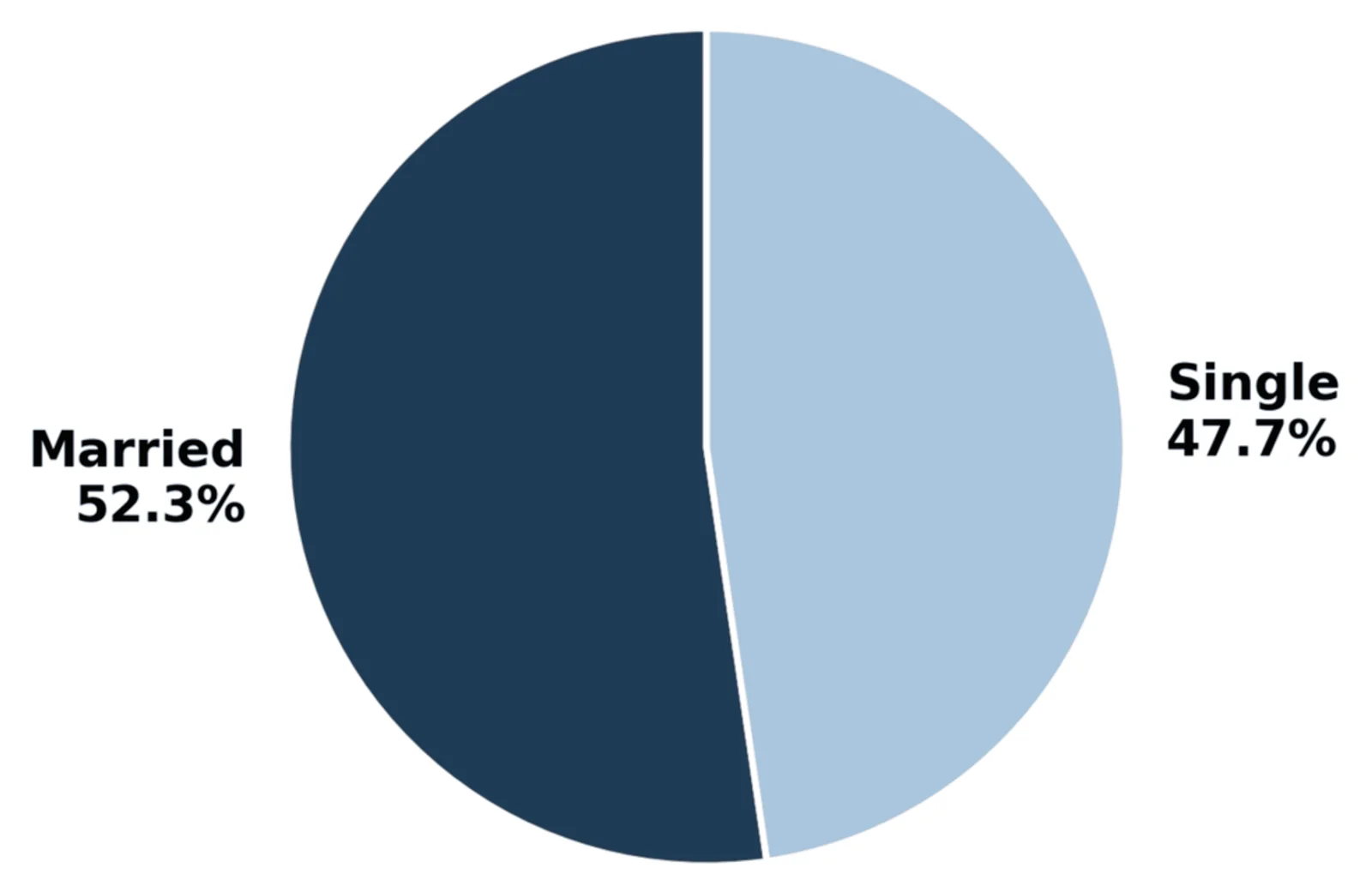
Marital status of the participants (N = 281)

**Table 2:** Age distribution of the participants (N = 281)

Variable	Category	N	%
Age (years)	18-29	100	35.6
	30-39	100	35.6
	40-49	51	18.1
	50-60	30	10.7
Total		281	100

**Figure 4:**
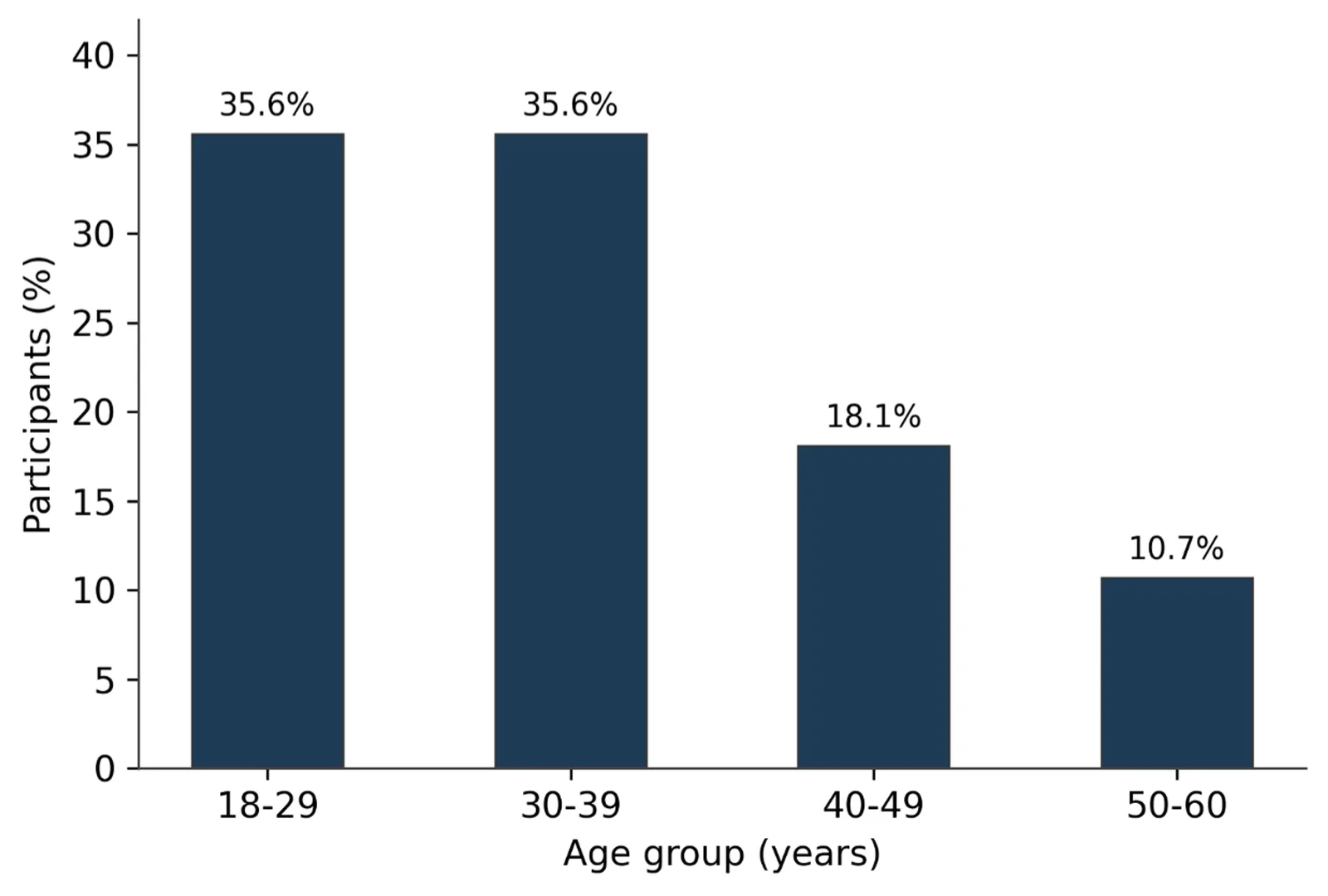
Age distribution of the participants (N = 281)

Health-related characteristics and symptom recognition

Approximately one-quarter of participants (23.8%) reported having a chronic disease, and 33.1% reported a family history of a heart attack (Table 3). Recognition of individual MI symptoms varied considerably, with typical symptoms recognized more frequently than atypical symptoms. Shortness of breath (76.9%) and chest pain (75.8%) were the most commonly recognized symptoms, followed by pain or heaviness in the left arm (73.3%) and sudden excessive sweating (70.5%). Recognition was notably lower for jaw or neck pain (54.8%) and nausea or vomiting (50.9%).

**Table 3:** Health-related data and recognition of MI symptoms (N = 281) MI: myocardial infarction

Variable	Category	N	%
Chronic disease	No	214	76.2
	Yes	67	23.8
Family history of heart attack	Yes	93	33.1
	No	121	43.1
	Don't know	67	23.8
Chest pain	Yes	213	75.8
	No	37	13.2
	Don't know	31	11
Shortness of breath	Yes	216	76.9
	No	34	12.1
	Don't know	31	11
Pain/heaviness in left arm	Yes	206	73.3
	No	38	13.5
	Don't know	37	13.2
Jaw or neck pain	Yes	154	54.8
	No	59	21
	Don't know	68	24.2
Nausea or vomiting	Yes	143	50.9
	No	78	27.8
	Don't know	60	21.4
Sudden excessive sweating	Yes	198	70.5
	No	44	15.7
	Don't know	39	13.9
Total		281	100

Recognition of typical MI symptoms (objective 1)

Recognition of the three typical MI symptoms was high overall: shortness of breath (76.9%), chest pain (75.8%), and pain/heaviness in the left arm (73.3%) were each recognized by roughly three-quarters of participants (Table 3). At the participant level, 176 of the 281 participants (62.6%) correctly recognized all three typical symptoms, 43 (15.3%) recognized exactly two, 21 (7.5%) recognized exactly one, and 41 (14.6%) recognized none of the three typical symptoms (Table 4).

**Table 4:** Distribution of participants by number of typical MI symptoms correctly recognized (N = 281) Values computed from participant-level responses across the three typical symptom items (chest pain, shortness of breath, pain/heaviness in the left arm) MI: myocardial infarction

Number of typical symptoms recognized (0-3)	N	%	Cumulative %
0	41	14.6	14.6
1	21	7.5	22.1
2	43	15.3	37.4
3 (All typical symptoms recognized)	176	62.6	100
Total	281	100	100

Recognition of atypical MI symptoms (objective 2)

Recognition was notably lower for two of the three atypical symptoms: jaw or neck pain was recognized by 154/281 participants (54.8%), and nausea or vomiting by 143/281 participants (50.9%). Sudden excessive sweating, though technically atypical, was recognized more often, by 198/281 participants (70.5%) (Table 3, Figure 5). All recognition rates were calculated using the full analytic sample (N = 281) as the denominator, since there were no missing responses on these items.

**Figure 5:**
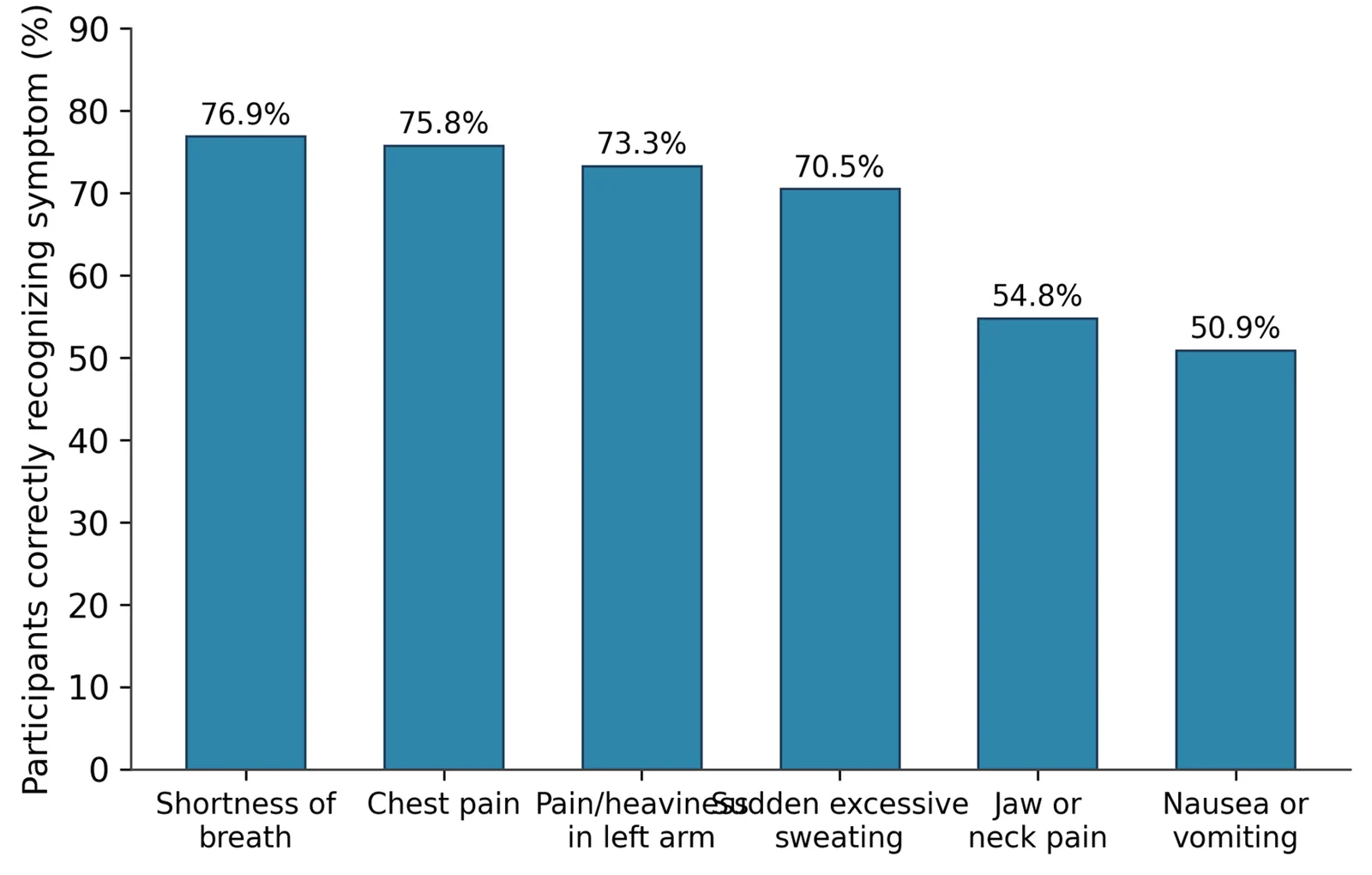
Recognition of MI symptoms (% answering “Yes”) MI: myocardial infarction

Appropriate first action (objective 3)

The great majority of participants (94.0%, 264/281) correctly identified calling an ambulance as the appropriate first action upon witnessing MI symptoms, supporting the interpretation that behavioral knowledge of the correct emergency response is strong, even where symptom-recognition knowledge, particularly for atypical presentations, is comparatively weaker. Smaller proportions incorrectly reported going to the nearest pharmacy (3.6%), waiting until symptoms disappear (1.8%), or taking painkillers (0.7%) (Table 5, Figure 6).

**Table 5:** Awareness of the appropriate emergency response to suspected MI (N = 281) MI: myocardial infarction

Variable	Category	N	%
First action when witnessing MI symptoms	Call an ambulance	264	94
	Go to the nearest pharmacy	10	3.6
	Take painkillers	2	0.7
	Wait until symptoms disappear	5	1.8
Delaying the hospital visit is dangerous	Yes	261	92.9
	No	14	5
	Don't know	6	2.1
Safe waiting time before seeking help	Immediately, within minutes	250	89
	Within an hour	10	3.6
	Can wait several hours	2	0.7
	Don't know	19	6.8
Ambulance better than private transport	Yes	255	90.7
	No	16	5.7
	Don't know	10	3.6
Emergency number in Saudi Arabia	911 (correct)	187	66.5
	997	75	26.7
	Other/incorrect	19	6.8
Total		281	100

**Figure 6:**
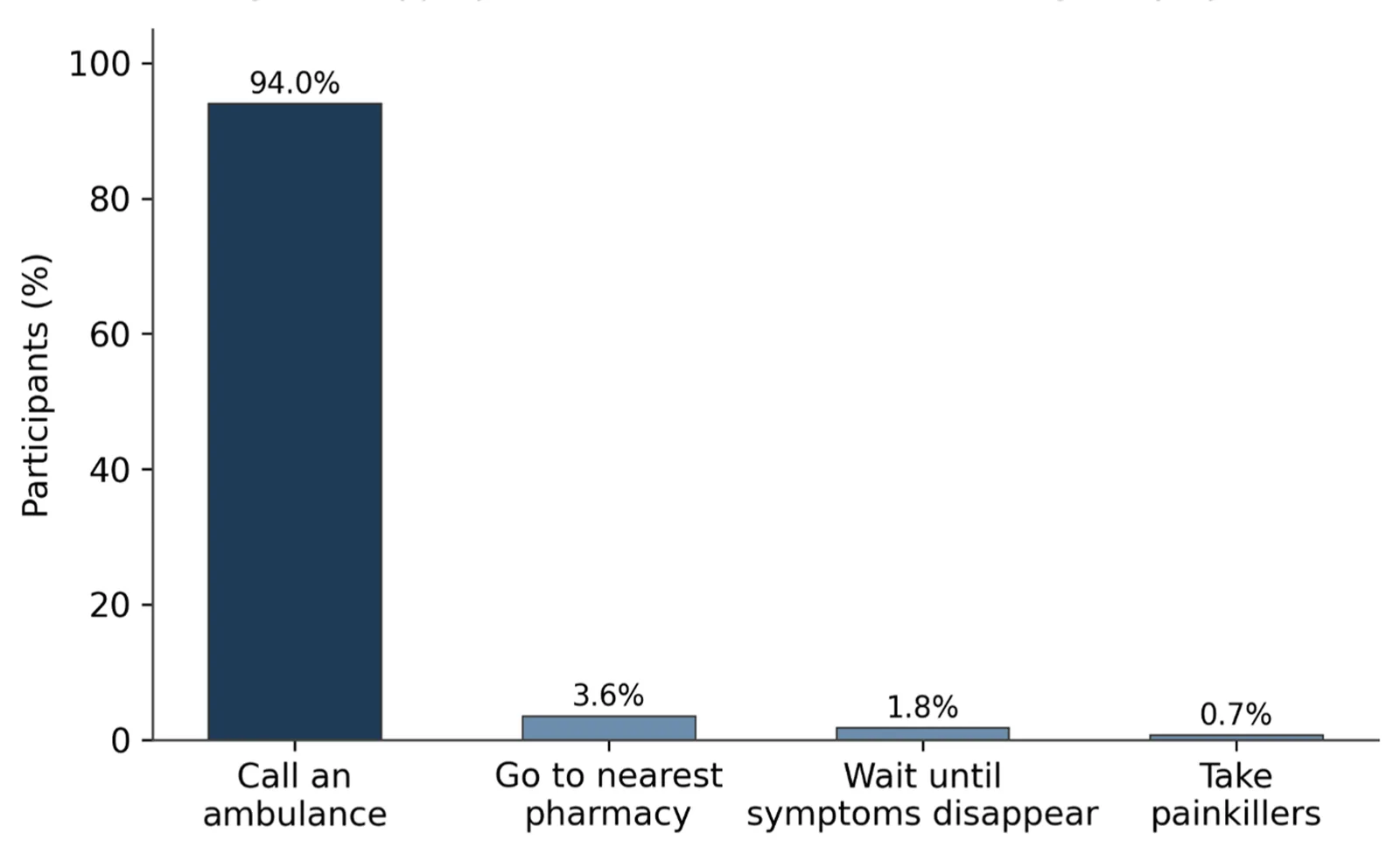
Appropriate first action when witnessing MI symptoms MI: myocardial infarction

Most participants recognized that delaying a hospital visit is dangerous (92.9%) and that immediate action, within minutes, is required (89.0%). Similarly, 90.7% agreed that calling an ambulance is preferable to private transport. Regarding the national emergency number, 66.5% of participants correctly identified 911, while 26.7% incorrectly reported 997 (the former Civil Defense number), and 6.8% gave another incorrect response.

Overall awareness score

An overall awareness score (range 0-6) was computed for each participant based on their correct recognition of the six listed MI symptoms. The mean awareness score was 4.02 ± 2.02 (median = 4), indicating that, on average, participants correctly recognized approximately four of the six symptoms, reflecting a generally good, though incomplete, level of symptom awareness in this population.

Sociodemographic and health-related determinants (objective 4)

Participants were classified as having high awareness (recognizing ≥ 4/6 symptoms; 66.9%, n = 188) or low awareness (recognizing ≤ 3/6 symptoms; 33.1%, n = 93). Chi-square tests showed no statistically significant association between awareness level and gender (χ² = 0.44, p = 0.506), educational level (χ² = 3.52, p = 0.318), or age group (χ² = 0.52, p = 0.914) (Tables 6-8, Figure 7).

**Table 6:** Distribution of study subjects according to awareness level and gender χ² = 0.44, p = 0.506. Since p > 0.05, there is no statistically significant relationship between gender and awareness level of MI symptoms MI: myocardial infarction

Gender	Category	High awareness		Low awareness		Total (N)
		N	%	N	%	
Gender	Male	78	69.6	34	30.4	112
	Female	110	65.1	59	34.9	169
	Total	188	66.9	93	33.1	281

**Table 7:** Distribution of study subjects according to awareness level and age group χ² = 0.52, p = 0.914. Since p > 0.05, there is no statistically significant relationship between age group and awareness level of MI symptoms MI: myocardial infarction

	Category	High awareness		Low awareness		Total (N)
		N	%	N	%	
Age (years)	18-29	66	66	34	34	100
	30-39	67	67	33	33	100
	40-49	36	70.6	15	29.4	51
	50-60	19	63.3	11	36.7	30
	Total	188	66.9	93	33.1	281

**Table 8:** Distribution of study subjects according to awareness level and educational level χ² = 3.52, p = 0.318. Since p > 0.05, there is no statistically significant relationship between educational level and awareness level of MI symptoms MI: myocardial infarction

	Category	High awareness		Low awareness		Total (N)
		N	%	N	%	
Educational level	High school	47	62.7	28	37.3	75
	Diploma	23	57.5	17	42.5	40
	Bachelor's	99	71.2	40	28.8	139
	Postgraduate	19	70.4	8	29.6	27
	Total	188	66.9	93	33.1	281

**Figure 7:**
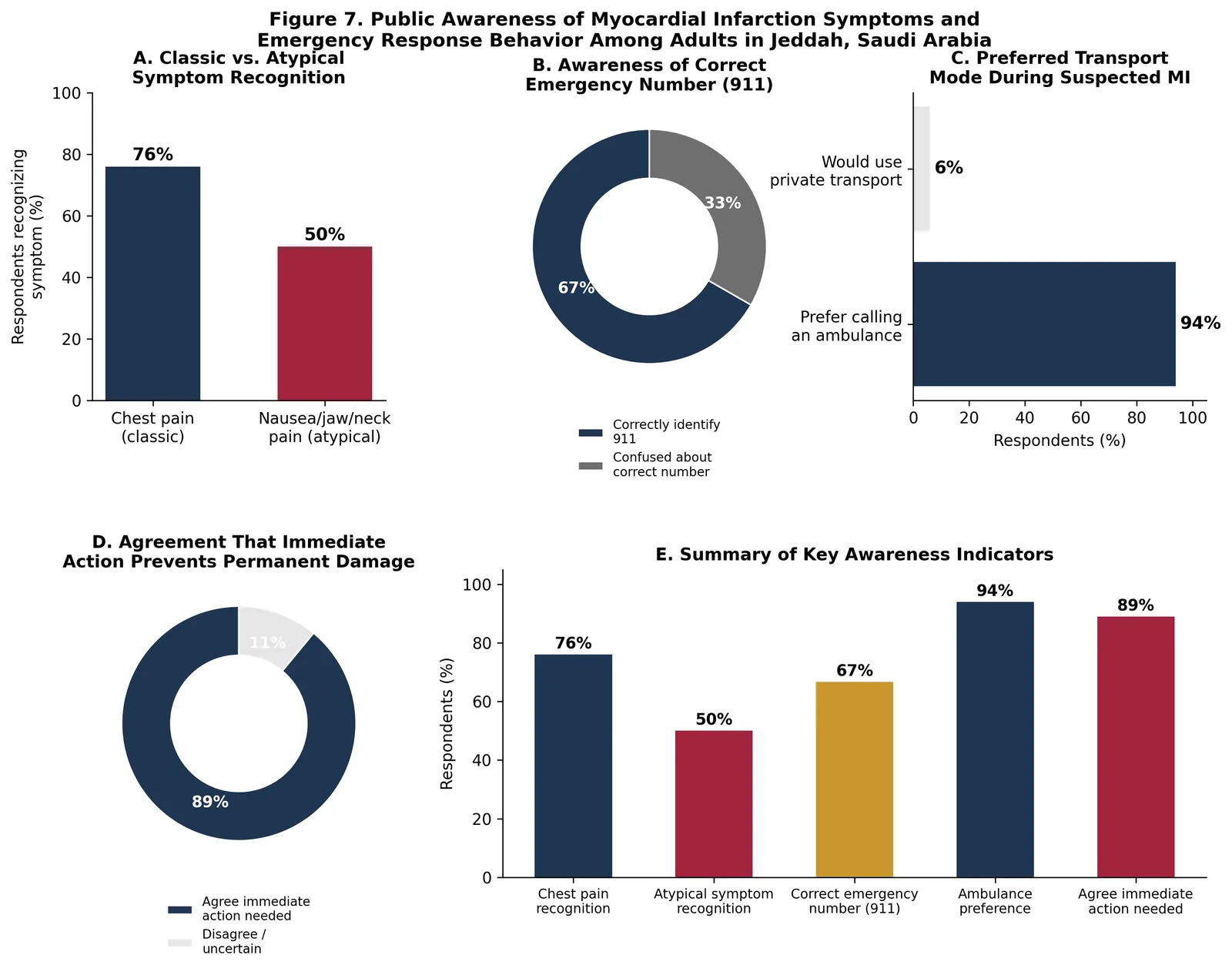
Results infographic Created by the authors using standard data visualization software (Python/Matplotlib)

Given that the achieved sample (N = 281) falls below the calculated target of 385, these non-significant findings should be interpreted as evidence that no large associations were detected in this sample, rather than definitive evidence that no true associations exist; the study was better powered to estimate overall prevalence than to detect small-to-moderate subgroup differences (see Limitations).

Comparison of mean awareness scores

An independent-samples t-test showed no statistically significant difference in mean awareness score between male (4.15 ± 1.97) and female (3.93 ± 2.05) participants (t = 0.88, p = 0.379) (Table 9). One-way ANOVA likewise showed no statistically significant difference in mean awareness score across educational-level groups (F = 0.57, p = 0.633) (Table 10) or across age groups (F = 0.34, p = 0.799) (Table 11).

**Table 9:** Comparison of mean awareness score by gender (independent-samples t-test) Since p > 0.05, there is no statistically significant difference in mean awareness score between males and females SD: standard deviation

Group	N	Mean	SD	t value	P-value
Male	112	4.15	1.97	0.88	0.379
Female	169	3.93	2.05		

**Table 10:** Comparison of mean awareness score by educational level (one-way ANOVA) Since p > 0.05, there is no statistically significant difference in mean awareness score across educational-level groups ANOVA: analysis of variance; SD: standard deviation

Group	N	Mean	SD	F value	P-value
High school	75	4	2.21	0.57	0.633
Diploma	40	3.75	1.96		
Bachelor's	139	4.04	1.91		
Postgraduate	27	4.41	2.14		

**Table 11:** Comparison of mean awareness score by age group (one-way ANOVA) Since p > 0.05, there is no statistically significant difference in mean awareness score across age groups ANOVA: analysis of variance; SD: standard deviation

Group	N	Mean	SD	F value	P-value
18-29	100	3.94	2.05	0.34	0.799
30-39	100	3.95	1.88		
40-49	51	4.24	2.14		
50-60	30	4.17	2.2		

## Discussion

This cross-sectional study assessed awareness of MI symptoms and the appropriate emergency response among 281 adults in Jeddah, Saudi Arabia. Overall, participants demonstrated a moderately good level of awareness, correctly recognizing an average of four of the six listed MI symptoms, with high awareness (≥ 4/6 symptoms) present in two-thirds of the sample. Consistent with prior research from Saudi Arabia and other settings, recognition of classic MI symptoms, particularly shortness of breath and chest pain, was considerably higher than recognition of atypical symptoms such as nausea or vomiting and jaw or neck pain [4-6,8,11,12]. This pattern likely reflects the prominence of chest pain and dyspnea in public health messaging and popular media portrayals of heart attacks, which may leave the public less equipped to recognize presentations that deviate from this classic picture. Because atypical presentations are disproportionately common among women, older adults, and people with diabetes, low awareness of these symptoms may be an important, modifiable contributor to delayed care-seeking in these groups [4,5,7,19].

Encouragingly, awareness of the appropriate emergency response was high: the vast majority of participants correctly identified calling an ambulance as the first action, recognized the danger of delaying hospital presentation, and understood that immediate action within minutes is required. This finding is broadly consistent with other Saudi studies reporting good general knowledge of appropriate emergency action despite gaps in specific symptom recognition [10,12,14], although it contrasts with a lower rate of correct emergency-response identification reported among adults in Alahssa [12], suggesting that awareness of appropriate action may still vary meaningfully by region within the Kingdom. Notably, roughly one in four participants incorrectly identified 997 rather than 911 as the emergency number, suggesting some residual confusion likely related to the former Saudi Civil Defense number; this represents a straightforward, correctable target for public messaging.

In contrast to some previous studies that reported higher awareness among more educated or higher-income individuals [8,9,17,18], this study found no statistically significant association between awareness level (or mean awareness score) and educational level, gender, or age group. This finding suggests that, in this sample, symptom awareness may be relatively evenly distributed across sociodemographic strata, which is somewhat reassuring from a health-equity perspective but also indicates that broad, population-wide awareness campaigns, rather than campaigns narrowly targeted at specific sociodemographic subgroups, may be the most efficient strategy for improving MI symptom recognition in this setting. The absence of significant associations may also reflect the relatively homogeneous, largely internet-literate nature of participants recruited through social media, which may have attenuated differences that would be more apparent in a more representative, population-based sample; low health literacy has separately been linked to poorer understanding of health information and poorer health outcomes more broadly, and may be an unmeasured confounder in studies of this kind [29].

These findings are broadly consistent with regional literature. Alsaab et al. reported similarly variable awareness of atypical MI symptoms and risk factors among Saudi adults, alongside a positive association between education and awareness that was not replicated here [8], while Elsherbiny et al. and Basham et al. reported comparable gaps between recognition of typical and atypical symptoms in other Saudi populations [10,11]. Taken together with the present findings, this suggests that the pattern of stronger awareness of classic symptoms alongside weaker recognition of atypical presentations may be a relatively consistent feature of public MI knowledge across different regions of the Kingdom [8,10-22], reinforcing the case for a national, rather than purely local, public health response, consistent with priorities articulated for cardiovascular disease prevention under Saudi Vision 2030 [23].

Limitations

Several limitations should be considered when interpreting these findings. First, the use of online, snowball sampling via social media [26] may have introduced selection bias, likely over-representing individuals with higher digital literacy, internet access, and educational attainment, which may limit the generalizability of findings to the broader Jeddah population, including older adults and those with limited internet access. Second, the self-administered questionnaire relied on self-reported knowledge, which may be subject to social desirability bias, whereby participants over-report their awareness to present themselves favorably. Third, the achieved sample size (281) was below the calculated target of 385 (73% of target); recalculating the margin of error for the achieved sample at the same 95% confidence level and P = 0.5 gives approximately 5.85%, versus the 5.00% originally targeted. This modest reduction in precision is judged acceptable for the overall prevalence estimates reported here, but the subgroup comparisons (chi-square, t-test, ANOVA) are correspondingly more likely to be underpowered to detect small effect sizes, and the reported non-significant associations should be interpreted with this limitation in mind. Finally, the cross-sectional design precludes causal inferences about the determinants of MI awareness.

## Conclusions

Awareness of classic MI symptoms and the appropriate emergency response was generally good among adults in Jeddah, Saudi Arabia, with the vast majority correctly identifying calling an ambulance as the appropriate first action. However, recognition of atypical symptoms, particularly nausea or vomiting and jaw or neck pain, remained comparatively low, and this gap was not explained by the sociodemographic and health-related factors examined. These findings support the need for broad-based, population-wide public health campaigns that specifically emphasize atypical MI presentations and reinforce accurate knowledge of the national emergency number, rather than campaigns narrowly targeted at specific demographic subgroups.

## Appendices

Study questionnaire: awareness of myocardial infarction symptoms and emergency response

Questionnaire Item

Response Options

Section A: Sociodemographic and Health Background

1. Age

18-29 / 30-39 / 40-49 / 50-60

2. Gender

Male / Female

3. Education level

Secondary / Diploma / Bachelor's degree / Postgraduate level

4. Marital status

Single / Married

5. Do you suffer from any chronic diseases?

Yes / No

6. If yes, please select:

Diabetes / Hypertension (high blood pressure) / Heart disease / High cholesterol

7. Is there anyone in your family who has had a heart attack?

Yes / No / I don't know

Section B: Recognition of Heart Attack Symptoms - "Do you think the following symptoms could be signs of a heart attack?"

8. Chest pain?

Yes / No

9. Shortness of breath?

Yes / No / I don't know

10. Pain or heaviness in the left arm?

Yes / No / I don't know

11. Pain in the neck or jaw?

Yes / No / I don't know

12. Nausea or vomiting?

Yes / No / I don't know

Section C: Emergency Response and Help-Seeking Behavior

13. If you feel or see someone with symptoms of a heart attack, what is the first action that should be taken?

Call an ambulance immediately / Wait until the symptoms go away / Take painkillers

14. Do you think delaying going to the hospital when heart attack symptoms occur could put the patient's life at risk?

Yes / No / I don't know

15. What do you think is the safe amount of time to wait before seeking help when heart attack symptoms appear?

Immediately, within minutes / Within one hour / It is possible to wait several hours / I don't know

16. What is the emergency number in the Kingdom of Saudi Arabia?

Open-ended (free text)

Note: The questionnaire was administered in both Arabic and English. Response options for items 9-12 and 14-16 included an "I don't know" option in addition to "Yes/No" to capture uncertainty.
